# Misincorporation by RNA polymerase is a major source of transcription pausing *in vivo*

**DOI:** 10.1093/nar/gkw969

**Published:** 2016-10-24

**Authors:** Katherine James, Pamela Gamba, Simon J. Cockell, Nikolay Zenkin

**Affiliations:** 1 Centre for Bacterial Cell Biology, Institute for Cell and Molecular Bioscience, Newcastle University, Baddiley-Clark Building, Richardson Road, Newcastle Upon Tyne, NE2 4AX, UK; 2 Bioinformatics Support Unit, Newcastle University, William Leech Building, Framlington Place, Newcastle Upon Tyne, NE2 4HH, UK

## Abstract

The transcription error rate estimated from mistakes in end product RNAs is 10^−3^–10^−5^. We analyzed the fidelity of nascent RNAs from all actively transcribing elongation complexes (ECs) in *Escherichia coli* and *Saccharomyces cerevisiae* and found that 1–3% of all ECs in wild-type cells, and 5–7% of all ECs in cells lacking proofreading factors are, in fact, misincorporated complexes. With the exception of a number of sequence-dependent hotspots, most misincorporations are distributed relatively randomly. Misincorporation at hotspots does not appear to be stimulated by pausing. Since misincorporation leads to a strong pause of transcription due to backtracking, our findings indicate that misincorporation could be a major source of transcriptional pausing and lead to conflicts with other RNA polymerases and replication in bacteria and eukaryotes. This observation implies that physical resolution of misincorporated complexes may be the main function of the proofreading factors Gre and TFIIS. Although misincorporation mechanisms between bacteria and eukaryotes appear to be conserved, the results suggest the existence of a bacteria-specific mechanism(s) for reducing misincorporation in protein-coding regions. The links between transcription fidelity, human disease, and phenotypic variability in genetically-identical cells can be explained by the accumulation of misincorporated complexes, rather than mistakes in mature RNA.

## INTRODUCTION

Correct copying of genetic information into RNA is one of the requirements of successful gene expression. Overall transcription fidelity, i.e. correctness of the final RNA product, has an estimated error rate of ∼10^−3^–10^−5^ (1–5), and is a result of the accuracy of nucleotide incorporation by RNA polymerase (RNAP) and of the proofreading of occasional misincorporation events. The accuracy of nucleotide triphosphate (NTP) choice is mainly determined by the RNAP active site (5,6). Different misincorporations are not equally frequent (5), and sequencing of transcripts produced *in vitro* by *E. coli* RNAP has revealed a strong bias in errors toward G>A misincorporation (misincorporation of AMP instead of GMP, resulting in A:C mismatched base pair), with a C preceding (C_-1_ in the RNA) the misincorporation position (2).

**Figure 1.**
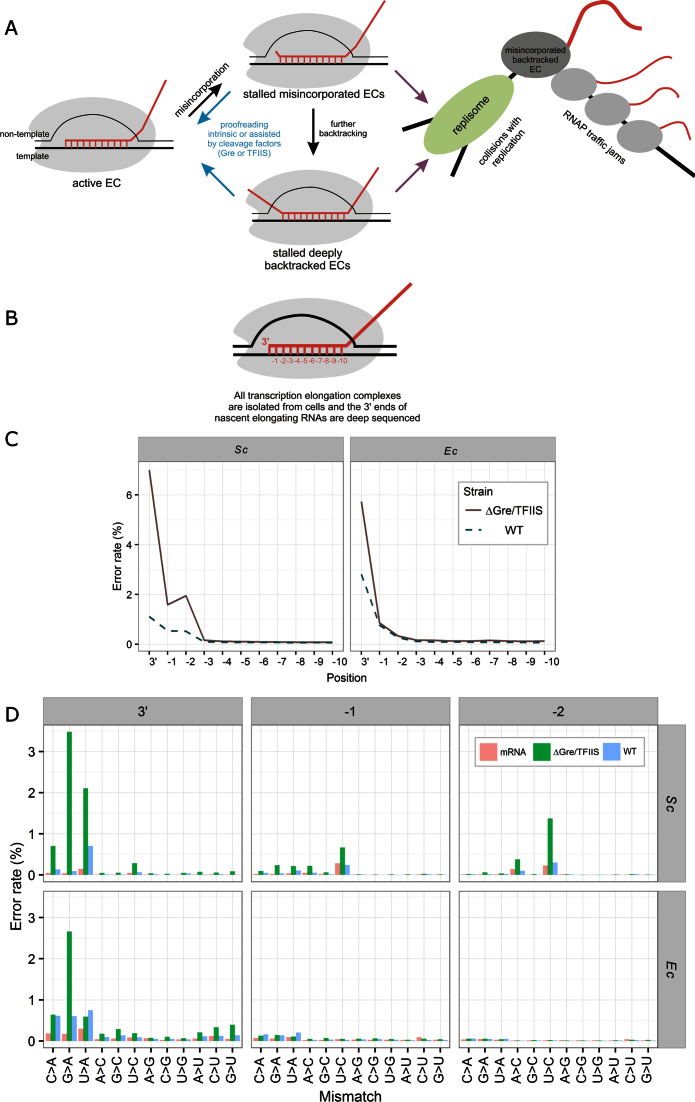
(**A**) Upon misincorporation, the elongation complex (EC) backtracks by 1 base pair, which then leads to further backtracking (7,8). Misincorporated and deeply backtracked ECs result in long-living pauses of transcription until resolved by intrinsic or factor-dependent cleavage. The paused ECs may cause collisions with replication, and cause RNAP traffic jams. (**B**) Native Elongating Transcripts sequencing (NET-seq) is a technique that involves sequencing of the 3΄ proximal parts of transcripts that are bound to transcribing RNAP. Shown is the scheme of the transcription EC, with positions in the transcript RNA (red) numbered from the 3΄ end. (**C**) The error rates at the 3΄ to –10 positions of the nascent RNAs of all active ECs with no filtering from *S. cerevisiae* (*Sc*; wild-type and ΔTFIIS mutant strains) and *E. coli* (*Ec*; independent data set for wild-type and ΔGre mutant strains). (**D**) The specific misincorporation rates at the 3΄, −1 and −2 positions for all ECs with no filtering from wild-type and mutant *E. coli* and *S. cerevisiae* strains.

Upon misincorporation, the elongation complex (EC) backtracks by 1 base pair (7,8) (scheme in Figure 1A). From this conformation, the hydrolysis of the second phosphodiester bond of the transcript by the RNAP active center removes the error in the form of a dinucleotide (8). *In vitro*, this reaction is greatly stimulated by cleavage factors, Gre for bacterial RNAP (9) and TFIIS for eukaryotic RNAP II (10). Besides contributing to errors in the final RNA products, misincorporation events were shown to cause long-lived pausing due to RNAP backtracking *in vitro* (7,8). However, until recently, the misincorporation-caused backtracked pauses have not been investigated *in vivo* due to their random and transient nature. Additionally, misincorporation-induced pauses were overlooked due to the small effect of cleavage factors on the error rate in the final RNA products (2,11), which led to the intuitive suggestion that misincorporation is a very rare event and, thus, could not contribute to pausing significantly. Importantly, however, backtracked pauses can be detrimental to cells; for instance, sequence-specific backtracked pauses have been proposed to cause RNA polymerase traffic jams, and were shown to cause conflicts with replication forks, leading to DNA double-strand breaks and genome instability (11–13).

An increase in transcription error rate has been linked to aging and various diseases (14–17), and may be a significant source of stochastic variability at the single-cell level (1,18). The generally accepted view is that the effects of lower transcriptional fidelity on cellular functions are caused by the mistakes in final RNA products, despite the error rate of mRNA translation being much higher than that of transcription. In contrast, the possible involvement of misincorporation-caused pausing is usually not considered, since it is thought to be a rare event. However, while overall fidelity can be reliably measured *in vivo*, the dynamics of the formation and resolution of misincorporated complexes, and their possible roles in the cell, remain obscure.

Native Elongating Transcripts sequencing (NET-seq) is a technique that involves sequencing of the 3΄ proximal parts of transcripts that are bound to transcribing RNAP, i.e. are actively elongating (19) (scheme in Figure 1B). These data provide a global snapshot of all transcription elongation complexes in the cell with precise identification of the 3΄ ends of the nascent RNAs. NET-seq has been used to study genome-wide pausing of transcription by identifying those genomic positions enriched with the 3΄ ends of the nascent RNAs (19–22). Further analysis of ECs using an RNase footprinting technique showed that ∼1% of backtracked ECs in *E. coli* strain lacking Gre factors are misincorporated (20).

Here, we analyzed published NET-seq datasets of all (not limited to paused ECs) nascent elongating RNAs in *E. coli* and yeast strains with and without cleavage factors for possible errors at 3΄ positions (19,21). We found that a far higher proportion of all ECs (1–3% in wild-type and 5–7% in mutant strains) is represented by stalled misincorporated complexes than has been proposed. The analysis also suggests that, despite the sequence bias of misincorporation events, the pausing *per se* may not be required for an increased rate of misincorporation, in contrast to what has been suggested earlier (20).

## MATERIALS AND METHODS

### Data sources

NET-seq data for *Saccharomyces cerevisiae* and *E. coli*, for both wild type and *dst* or *greA*/*B* deletion strains, and equivalent total RNA-seq data (Supplementary Table S1) (19,21), were downloaded from the National Center for Biotechnology Information's (NCBI) Gene Expression Omnibus (23) website and converted into fastq format using sratoolkit version 2.5.2 (http://www.ncbi.nlm.nih.gov/sra). The reference genome for *E. coli* (NC_000913.3) was also downloaded from NCBI (23), while the nuclear *S. cerevisiae* reference genome (S288C) was downloaded from the Saccharomyces Genome Database (24).

### Quality control and pre-processing

Dataset quality was assessed using FastQC (http://www.bioinformatics.babraham.ac.uk/projects/fastqc/) to ensure per base and per tile sequence quality. Where necessary raw reads were adaptor trimmed as described in the original publications (19,21). In the case of the *S. cerevisiae* datasets, reads aligning to tRNAs, snoRNAs and rRNAs were removed as described by Weissman and colleagues (19). Reads from the *Ec*WT dataset with N bases at the 3΄ adjacent position were also excluded (21) due to a systematic sequencing error at this position identified during quality control (Supplementary Figure S1).

### Genome alignment

We employed a stringent genome alignment strategy in order to optimize the accuracy of the error rate calculation (Supplementary Figure S2). K-mer counts were performed using jellyfish at default settings over both strands (25). Reads were aligned to genomes using Bowtie (26) allowing two mismatched bases in a seed region of 14 where only unique alignments were reported (-n 2 –l 14 –m 1).

### Error rates

Data analysis was carried out in R using the BioConductor seqTools (27) and IRanges (28) packages. Total error rates were calculated as the percentage of total reads with a mismatched base at each read position in the alignment, thresholded to a Phred quality score of <30 (Supplementary Figure S3) and excluding mismatches involving ambiguous *N* bases. Specific error rates were calculated as the percentage of total reads with a specific mismatch, for example an A incorporated instead of a G (G > A misincorporation), at each read position, thresholded to a Phred quality score of <30 and excluding mismatches involving ambiguous *N* bases.

### Statistical analysis

Experimental rates of false positives (matches classed as mismatched) and false negatives (mismatches classed as matches) were calculated based on the published error rates for the enzymes used in the reverse transcriptase (Primerscript (Clontech.com) or Superscript (29)) and PCR (PrimeStar Max (Clontech.com) or Phusion (https://www.neb.com/faq)) steps, and for the sequencing miscall rate of 1 in 1000 based on a Phred score threshold of 30 (30). In all cases there was assumed to be a two in three chance of a genuine mismatch remaining mismatched following an error. Accuracy of the error rates was then calculated as the percentage of all observed errors that were true positive mismatches.

### Sequence analysis

Single base variations between the experimental strains and their reference genomes were identified using samtools and bcftools following the method of Li (31,32). The positions of specific misincorporations (for instance G>A) were then mapped to the reference genomes using the BioConductor seqTools (27) and IRanges (28) packages, excluding those at positions with identified mutations. Misincorporation hotspots for the *Ec*ΔGre and *Sc*ΔTFIIS datasets were defined as having >50 misincorporations. Sequence logos were created using the R seqLogo package (33).

Generic Feature Format Version 3 files (GFF3) were downloaded from the NCBI website in order to identify protein coding regions (CDSs). Aligned locations were identified from the bowtie output using BEDTools (34), and the BioConductor seqTools (27) and IRanges (28) packages. Transcribed but non-translated regions (UTRs) in *Ec*RNA were identified using Rockhopper (35), and an *S. cerevisiae* S288C UTRs (36) were obtained from the Saccharomyces Genome Database (24).

‘Translated’ regions were defined as aligned locations within CDSs, while ‘transcribed non-translated’ regions as aligned locations within the UTRs. In *S. cerevisiae* introns were also included in the ‘transcribed non-translated’ regions. Misincorporation rates were calculated for the *Ec*ΔGre and *Sc*ΔTFIIS genomic regions as misincorporated positions per 100 000 bp and as hotspots per 100 000 bp.

## RESULTS

We analyzed the NET-seq data for wild-type *S. cerevisiae* (*Sc*WT), a mutant *S. cerevisiae* lacking the cleavage factor TFIIS (*Sc*ΔTFIIS) (19), wild-type *E. coli* (*Ec*WT), and a mutant *E. coli* lacking cleavage factors GreA and GreB (*Ec*ΔGre) (21) (Supplementary Table S1).

To enable high accuracy error rate calculation, the data were subject to extensive bioinformatic pre-processing prior to alignment to reference genomes (Supplementary Figures S1 and S2). Equivalent mRNA-seq data (conventional sequencing of total RNA (19,21), available for the *Sc*WT (*Sc*RNA) and *Ec*WT (*Ec*RNA) datasets) were analyzed in parallel as a control for possible mistakes during library preparation and sequencing, and to account for differences between the laboratory strains and their reference genome sequences. We compared total error rates from the 3΄ end to position -10 of the aligned transcripts, which approximately corresponds to the length of the RNA/DNA hybrid within the EC (Figure 1B). Surprisingly, we found that the very 3΄ position of the nascent RNAs carried a large number of erroneous nucleotides in all strains (Figure 1C). The error rate at positions −1 to −10 was far lower and comparable to the overall error rate in the total RNA-seq data (Supplementary Figure S4, Table S2, see below regarding −1 and −2 positions of yeast data), indicating that 3΄ mismatched reads in the alignment represent the misincorporated ECs. The proportions of misincorporated ECs in *Ec*WT and *Sc*WT were approximately 3% and 1% of all ECs, respectively (Figure 1C). In mutant strains lacking cleavage factors *Ec*ΔGre and *Sc*ΔTFIIS, the proportions of misincorporated ECs were ∼5% and 7%, respectively (Figure 1C). Although consistent with the ability of cleavage factors to proofread misincorporation events, the proportions of misincorporated ECs in WT and mutant strains were much higher than one would expect given the error rate of synthesis by RNAP: 10^−3^–10^−6^ (5,6,37,38). The proportion of misincorporated ECs was also far higher than could be proposed based on the assumption that 1% of backtracked ECs are misincorporated (20).

**Figure 2.**
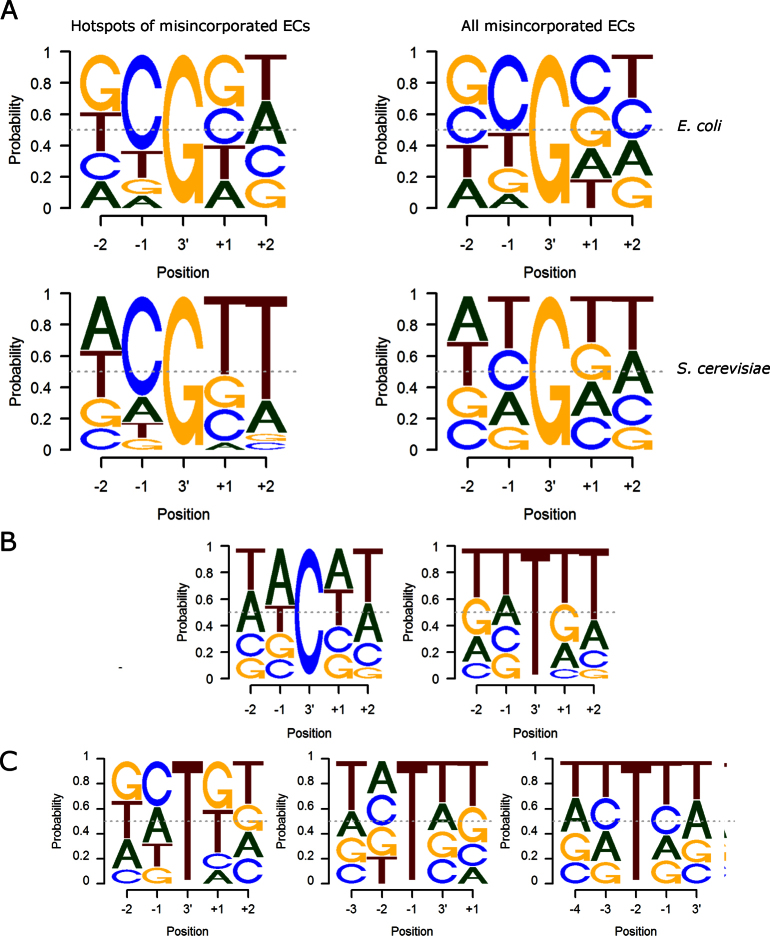
Sequence logos for the specific misincorporations (T of the read corresponds to U in the RNA). (**A**) The sequences surrounding the G>A misincorporations in the *Ec*ΔGre and *Sc*ΔTFIIS strains. (**B**) The sequences surrounding the C>A and U>A hotspots for the *Sc*ΔTFIIS strain. C. The sequences surrounding the U>C misincorporations at the 3΄, −1 and −2 positions in *Sc*ΔTFIIS.

The pattern of specific 3΄ misincorporations was similar for all datasets with a strong bias toward G>A misincorporations (Figure 1D), consistent with previous observations *in vitro* (2,5,38). There were several G>A misincorporation hotspots - positions where misincorporation happened frequently (>50 reads per location). For these hotspots in the mutant *E. coli* and *S. cerevisiae* there was a clear bias toward C preceding the position of the G>A misincorporation (Figure 2A), consistent with previous observations on final RNA products (2). The sequence bias in hotspots is also consistent with the earlier finding that CG motifs increase G>A misincorporations (20) (although we do not observe coincidence of misincorporation hotspots and pausing at −1 position), and suggests that this mechanism is conserved between bacteria and eukaryotes. However, the far largest number of G>A misincorporation events was away from hotspots, and represented by only one to few reads per location, suggesting that G>A misincorporation is a quite random event. When all misincorporation events were taken into account, the bias toward C preceding G>A misincorporation decreased in *E. coli* and disappeared in *S. cerevisiae* (Figure 2A), indicating that formation of many misincorporated ECs is not restricted to the CG motifs or pause sites.

*S. cerevisiae* also showed considerable U>A and C>A misincorporations at 3΄ ends (Figure 1D), though with less obvious sequence bias in the surrounding sequence of hotspots (Figure 2B). The error rates in the *S. cerevisiae* datasets were slightly elevated at positions −1 and −2, with bias toward U>C mismatch in both positions (Figure 1D). The sequences downstream of the −1 and −2 U>C misincorporations were slightly biased toward Us, suggesting that some misalignment of the template may favor read-through of misincorporated C by one or two positions (Figure 2C). It is also possible that U>C errors in the −1 and −2 positions of the transcript may facilitate pausing or backtracking of the EC, thus resulting in the accumulation of these mistakes in nascent transcripts. Another unexpected observation from the *S. cere*visiae misincorporated ECs was a bias toward Ts at the +1 and +2 positions in the non-template strand (irrespective of the misincorporated NMP; Figure 2C). While this bias could be due to a complex structural effect, it is also possible that this sequence diminishes the selection of correct NTPs by the template strand downstream of the active center proposed for eukaryotic RNAPs (39).

We found that in protein-coding sequences (ORFs) of *E. coli*, the G>A misincorporation hotspots were far less abundant than in the transcribed untranslated regions, with 1.34 and 10.68 hotspots per 0.1 Mb, respectively (Table 1). Other (non-hotspot) G>A misincorporated ECs were distributed evenly. No difference in distribution of hotspots or other misincorporated complexes between coding and non-coding regions were seen in *S. cerevisiae*, suggesting existence of a bacteria-specific mechanism to minimize formation of misincorporated ECs in protein coding sequences.

**Table 1.** Distribution of G>A misincorporations and hotspots

Dataset	Type	# locations	#ECs	Translated		Transcribed non-translated	
				Length (bp)	mm/100 000 bp	Length (bp)	mm/100 000 bp
*Sc*ΔTFIIS	All	197947	361319	7173492	1848.14	1 241 209	1326.21
	Hotspot	40	4939		0.22		0.24
*Ec*ΔGre	All	199307	519122	3871814	4356.2	140 405	4352.41
	Hotspot	223	35023		1.34		10.68

The number of G>A misincorporation (mm) positions and hotspots (G>A hotspots were defined as having >50 misincorporations) in the deletion mutants, and the misincorporation rates in the translated regions in comparison to the transcribed non-translated regions. Transcribed translated regions were defined as aligned locations within protein coding sequences, while transcribed non-translated regions as aligned locations within the untranslated regions. In *S. cerevisiae* introns were also included in the transcribed non-translated regions.

## DISCUSSION

Our bespoke pipeline was designed to optimize the accuracy of the error rate calculation while minimizing the loss of data and, consequently, the observed effects are likely to be biological, rather than experimental (library preparation, sequencing and data processing) in origin, for a number of reasons:

1. Although the reverse transcription step is known to introduce errors, since these enzymes do not proofread (40), and have estimated error rates in the order of 10^−4^ to 10^−5^ (41), the 3΄ error rate in the order of 10^−1^ to 10^−2^ observed here would not be affected by the documented error rates of the enzymes used in library preparation. In fact, error rates at the 3΄ position were estimated to be >70% non-experimental, while error rates in the −1 to −10 positions had far lower estimated accuracy (Supplementary Table S2). In addition, 3΄ error rates were significantly higher than error rates at randomly-generated 3΄ ends of the total RNA-seq controls (libraries originated from random alkaline fragmentation of total RNA) (Supplementary Figure S4).
2. The sequence specificity of misincorporation (G>A) was consistent with the bias observed in mature RNAs (2), and in nascent RNAs from a different study (20) (Figure 2A), and was different from the specificity of the errors in randomly-generated 3΄ ends of the control total RNA-seq datasets (libraries originated from random alkaline fragmentation of total RNA) (Figure 1D).
3. The observed difference in the 3΄ end error rate between WT and strains lacking proofreading factors would not be expected if these were experimentally-derived errors.
4. Assessment of dataset quality using FastQC (http://www.bioinformatics.babraham.ac.uk/projects/fastqc/) indicated sufficient quality to reliably quantify transcriptional fidelity using these data in the majority of cases, although some reads were omitted from the error rate calculations to ensure accuracy (Supplementary Figure S1).
5. Alignment was carried out allowing two mismatches within a seed region of 14 nucleotides, chosen to minimize seed length while ensuring k-mer uniqueness (Supplementary Figure S2). Alteration of the number of allowed mismatches has little effect on the alignments or observed error rate (Supplementary Figure S3A, Tables S3 and S4).
6. In order to reduce the effect of sequencing miscalls, a Phred threshold of 30, equivalent to a 99.9% base call accuracy rate (30), was applied at each position (Supplementary Figure S3B). Alteration of the Phred threshold also had virtually no effect on the observed error rates (Supplementary Figure S3C).
7. Positions with single base pair variations between experimental strain and reference genome were excluded from the positional analysis to ensure misincorporation rates were not inflated by genuine mutations.

An earlier study in *E. coli* analyzed 3΄ errors in NET-seq reads of particular lengths (14-18 nucleotides), representing ECs in different translocation states (20). This study found an unusually high rate (0.8%) of misincorporation associated with backtracked ECs in the ΔGre strain, which approximates to a misincorporation rate of <0.5% of total ECs in the cell (taking into account the distribution of misincorporations between translocation states, and possible underrating of the proportion of non-backtracked ECs). Using the NET-seq data from two different studies (19,21), we report an at least 10-fold higher abundance of misincorporated ECs in the mutant *E. coli* and *S. cerevisiae* strains lacking the proofreading factors. This difference in the proportion of misincorporated ECs can be explained by differences in the EC isolation protocols between the two studies. The DNA digestion preceding the ECs’ isolation was performed on ice in the studies by Larson *et al.* and Churchman *et al.* (19,21) (the data analyzed by us), which would slow down all the reactions of RNAP. In the study by Imashimizu *et al.* (20), this digestion procedure was performed at room temperature. We suggest that, at room temperature, the high concentrations of Mn^2+^ required by DNase I facilitated the intrinsic proofreading activity of RNAP, leading to lower proportions of misincorporated ECs. This variation in the NET-seq ECs’ preparation has also likely caused the striking difference in the proportions of the misincorporated ECs observed in the WT strains of these two studies; while our analysis of Larson *et al.* and Churchman *et al.* (19,21) data showed 1–3% of all complexes as misincorporated, Imashimizu *et al.* (20) reported ∼0.1%. We suggest that the difference was caused by ongoing Gre dependent proofreading during ECs isolation in the work by Imashimizu *et al*.

Based on their analysis of misincorporation within the subset of sequence-dependent pauses, Imashimizu *et al.* (20) suggested that, during sequence-dependent pausing, a C_-1_ increases the rate of G>A misincorporation at the following position (no sequence analysis was presented for non-paused ECs). This observation cannot exclude that misincorporation is induced merely by the C_-1_G_+1_ sequence, without involvement of a pause. Our analysis of all ECs did reveal several misincorporation hotspots with a clear bias to C preceding G>A misincorporation in both *E. coli* and *S. cerevisiae*. However, we did not observe any strong pausing at the position preceding misincorporation (−1 position; not shown), suggesting that these hotspots may occur at the misincorporation-inducing elements (C_−1_G_+1_) but without involvement of pausing.

**Figure 3.**
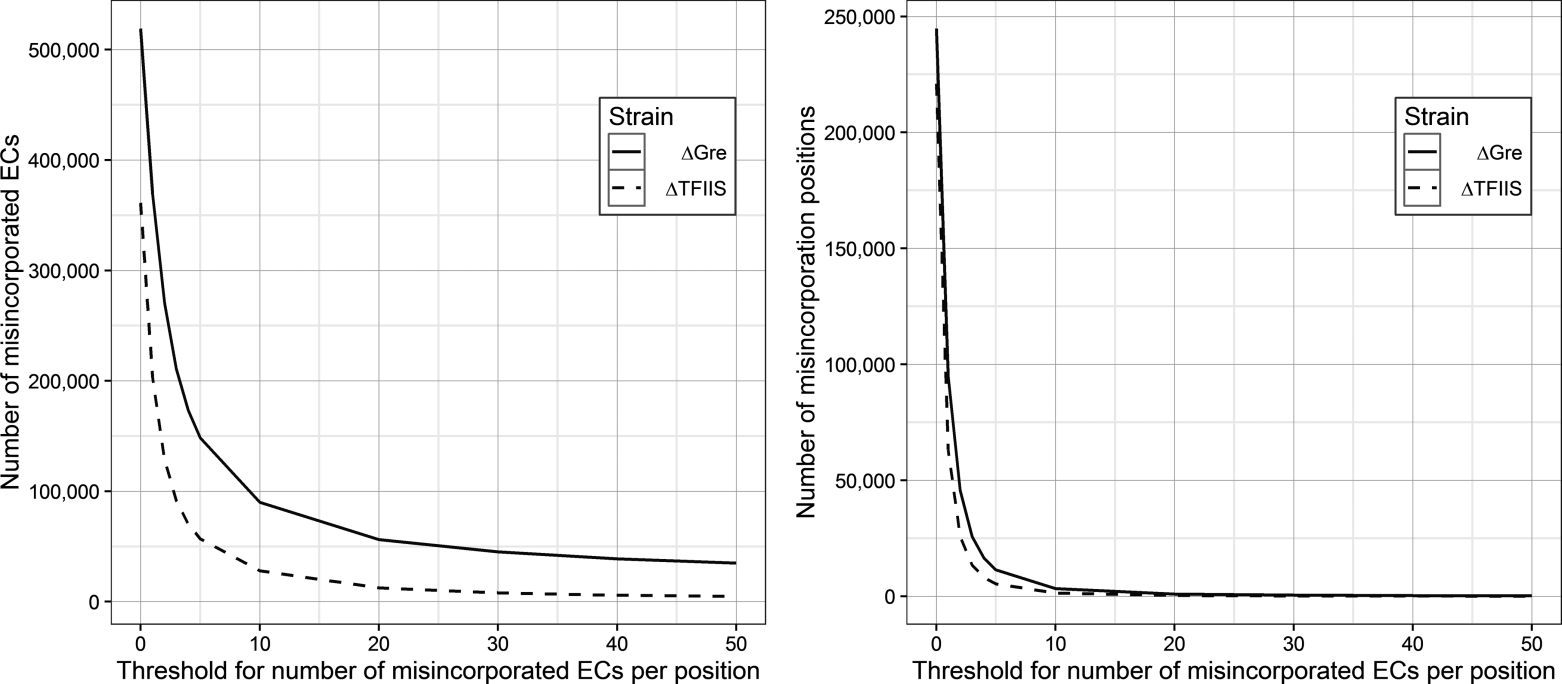
The number of misincorporated ECs (**A**) and misincorporation positions (**B**) as the threshold for misincorporations per position is increased. The vast majority of misincorporations occur at positions with a single misincorporation event.

Most of the misincorporations we observed were singular events (represented by one or few reads, Figure 3), indicating that they were not formed on particular misincorporation-inducing sequences, but happen more randomly. Furthermore, we observed lower (*E. coli*) or no (*S. cerevisiae*) sequence bias around the G>A misincorporations in the non-hotspot misincorporated ECs (Figure 2 A), which, thus, sequence-wise, also appear to be distributed more randomly (although in *E. coli* G>A misincorporation is somewhat favored at C_−1_G_+1_). Taken together, our results suggest an unusually high abundance of randomly distributed misincorporated ECs, and that misincorporation events may not be necessarily determined by the formation of a pause at C_−1_G_+1_ sequence prior to the misincorporation.

The sequence-bias toward G>A misincorporation, misincorporation hotspots at CG sequences, and the overall proportion of misincorporated ECs, were similar in *E. coli* and *S. cerevisiae* mutant and wild-type strains. This similarity indicates that the proposed pausing and misincorporation induced by CG sequences, and the overall mechanism of misincorporation and proofreading, are conserved between bacteria and eukaryotes. Misincorporation of A was more frequent than any other nucleotide, irrespective of the base in the template strand (Figure 1D). This observation could be explained by the presence of some structural or chemical determinants in the RNAP active site, which make binding of the non-complementary ATP less dependent on the template base and/or facilitate binding in the conformation favorable for misincorporation. Interestingly, the rate of the removal of the erroneous AMP (via transcript assisted second phosphodiester bond hydrolysis) is significantly faster than that for other mistakes (8,42). It is tempting to speculate that this propensity has evolved to counteract more frequent misincorporations of AMP.

Despite similarities in the mechanisms of misincorporation, the distribution of the misincorporation hotspots within the genome was different for *E. coli* and *S. cerevisiae* (Table 1). Hotspots were clearly reduced in protein-coding (1.34 hotspots per 0.1 Mb) versus transcribed but untranslated regions (10.68 hotspots per 0.1 Mb) in *E. coli*. It is possible that ORFs may have evolved to minimize error-prone sequences (hotspots) to reduce the formation of incorrect proteins. However, the absence of such bias in *S. cerevisiae* does not support this idea (Table 1). Instead, bacteria may have minimized the hotspots for formation of misincorporated ECs in the protein coding regions to reduce their interference with coupled translation, which would not be required in eukaryotes where transcription and translation are uncoupled. This idea is supported by the findings that sequence-specific pausing is also enriched in 5΄ untranslated regions (20). It is tempting to speculate that bacteria may have an as yet unrecognized mechanism(s) that decreases the formation of misincorporated ECs, or facilitates correcting/overcoming them, more efficiently at the error-prone sequences of ORFs than at other sequences and in the untranslated regions. One of the speculative possibilities could be that translation itself suppresses misincorporation events, although this hypothesis requires further investigation.

Misincorporation at some sequences has been shown to be much faster (∼10 times) than on random sequences (2). However, even these reported rates of misincorporation cannot account for the observed proportion of misincorporated ECs. Since misincorporation leads to stable backtracking (7,8), the observed proportion of misincorporated complexes is likely to be a result of their accumulation due to their inefficient resolution, even in the WT strains. In this scenario, misincorporated complexes accumulate relatively slowly, but are also slowly resolved. Importantly, the error rate in the mature RNA products would not be changed, since misincorporated ECs are not productive in formation of a mature RNA, until they are proofread. The high proportion of misincorporated ECs suggests that they could be a major source of strong pauses in the cell, and, thus, the main cause of conflicts with fellow RNAPs and replication complexes. This hypothesis is supported by the recent findings that DksA, which participates in prevention of collisions between transcription and replication (43), was found to increase the accuracy of RNA synthesis, i.e. decreases misincorporation events (44,45). It is also possible that random transient misincorporation pausing could be used by bacteria to help couple transcription and translation, by slowing down the former.

The very high proportion of misincorporated ECs observed here implies that one of the major roles of TFIIS and Gre factors is the resolution of misincorporation events. Since the input of cleavage factors into the correctness of the final RNA product is modest (2,4), our results suggest that the main function of the Gre and TFIIS factors is to physically resolve stalling of misincorporated ECs, rather than to correct the RNAs’ sequence *per se*. Indeed, a significant degree of cell filamentation, often accompanied by a diffuse nucleoid morphology, was observed in an *E. coli* mutant lacking Gre and DksA factors, indicative of problems with replication and/or chromosome segregation (not shown). Consistently, similar defects were observed in a Δ*greA* mutant of *S. pneumoniae*, which has only one Gre factor and has no DskA (11). Notably, factors involved in the repair of collapsed replication forks or double stranded breaks become essential in Δ*greA* Δ*greB* background (46). The mechanisms by which the cells deal with a high proportion of misincorporated ECs in the absence of the fidelity factors are the subject for future studies.

Stochastic fluctuations in protein expression, often referred to as noise, can cause significant phenotypic heterogeneity in isogenic cell populations and are essential for the activation of bimodal genetic switches that result in alternative expression states. It has been proposed that transcription errors could be a cause of such noise by leading to the production of non-functional regulatory proteins (18). However, our findings suggest that the random formation of misincorporated stalled ECs could also produce considerable noise by physically blocking transcription of regulatory genes. Similarly, it is possible that cellular defects and diseases linked to the fidelity of transcription (1,14–18) could, in fact, be caused by the accumulation of misincorporated ECs and subsequent conflicts with other molecular mechanisms, rather than by the correctness of the final RNA products *per se*.

## Supplementary Material

Supplementary Data
