# Supplementary material for: Misincorporation by RNA polymerase is a major source of transcription pausing in vivo
Source: Nucleic Acids Res. 2016 Oct 24;45(3):1105–13. doi: 10.1093/nar/gkw969 (PMC5388426; doi:10.1093/nar/gkw969)
Supplement: Supplementary Data [file gkw969_Supp.pdf]

Supplementary Information for:

Misincorporation by RNA polymerase is a major source of transcription pausing *in vivo*

Katherine James[1], Pamela Gamba[1], Simon J. Cockell[2], Nikolay Zenkin[1]

[1]Centre for Bacterial Cell Biology, Institute for Cell and Molecular Bioscience, Newcastle University, Newcastle upon Tyne, NE1 7RU, UK.
[2]Bioinformatics Support Unit, Newcastle University, Newcastle upon Tyne NE1 7RU, UK.

Contents:
Figure S1: Quality control.
Figure S2: Alignment strategy.
Figure S3: Parameterization.
Table S1: Data sources.
Table S2: Accuracy of error rates.
Table S3: *Saccharomyces cerevisiae* alignment statistics.
Table S4: *Escherichia coli* alignment statistics.

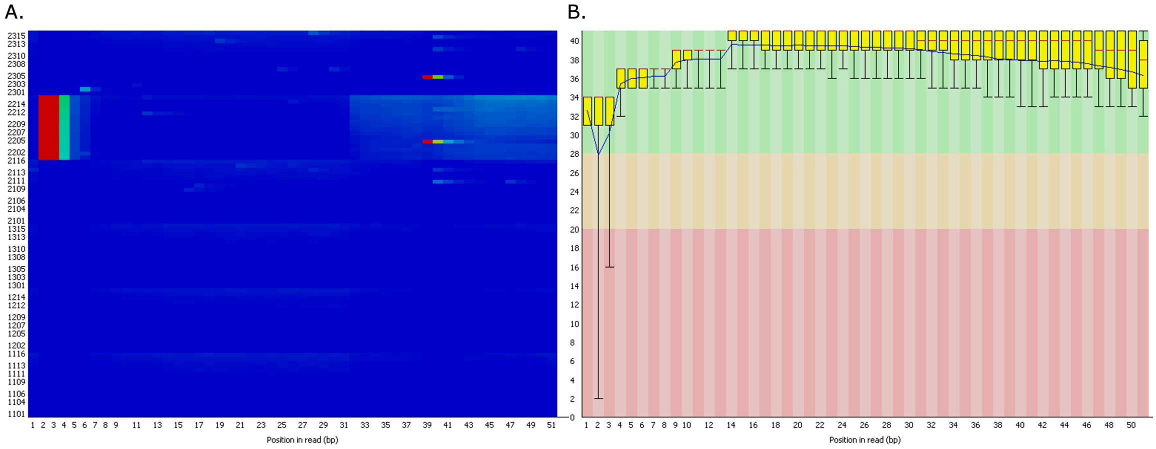

Figure S1: Quality control. Quality control of the *Ec*WT dataset using FastQC revealed a systematic error, likely to be caused by a bubble in the flowcell, adjacent to the 3' position in a number of reads. These reads (n = 5590806, 15.59%) were omitted from the error rate calculations. No systematic errors were identified in the other datasets. A. Example of quality scores from each tile across all bases at each position in the *Ec*WT reads. B. Example of quality values across all bases at each position in the *Ec*WT reads.

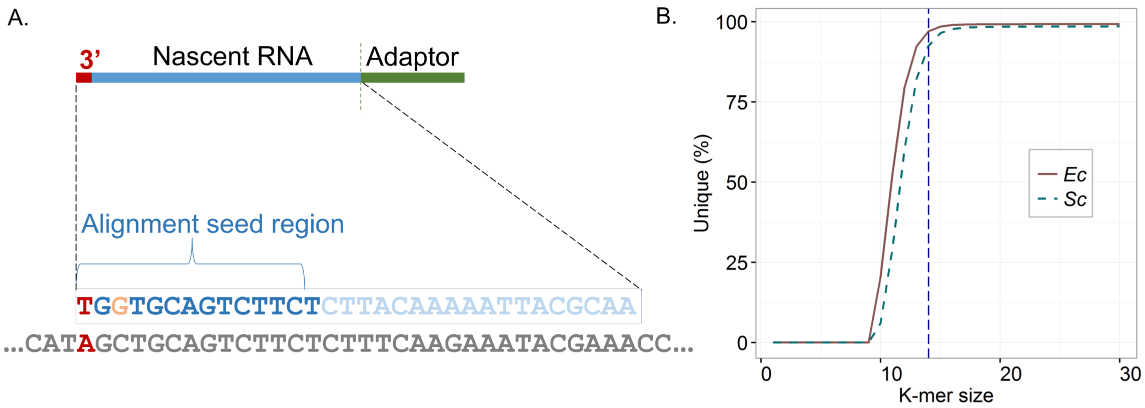

Figure S2: Alignment strategy. A. Alignment of the nascent RNA read was carried out following adaptor trimming allowing a maximum of two mismatches within the seed region. Error rates were calculated for all reads at each position. In this example an A>T mismatch, equivalent to A>U misincorporation in the nascent RNA, occurs at the 3' position, and a C>G mismatch at the -2 position. B. The seed region was chosen for alignment in order to minimize seed length, thus restricting mismatches to the region of the nascent 3', while ensuring seed uniqueness. A threshold of 14 (vertical blue dashed line) was chosen equivalent to $> 90\%$ uniqueness in all three genomes.

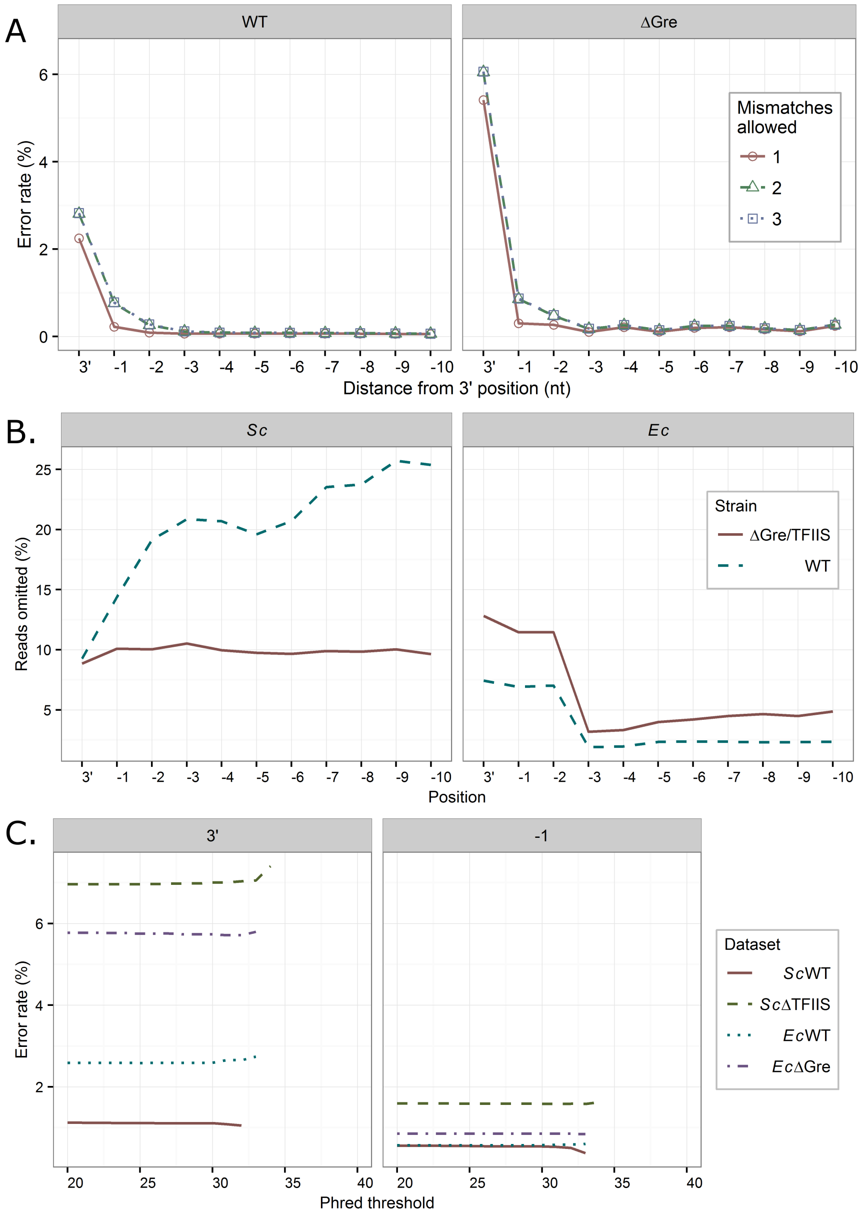

Figure S3: Parameterisation. Altering the number of mismatches allowed during alignment or the Phred quality threshold used for error rate calculation had little effect on the observed 3' error rates in all cases. A. The error rates for the EcWT and EcΔGre datasets allowing 1, 2 and 3 mismatches in the alignment. Reads were aligned to genomes using Bowtie using a seed region of 14 where only unique matches were reported. B. In order to reduce the effect of sequencing miscalls, a Phred threshold of 30, equivalent to a 99.9% base call accuracy rate, was applied at each position, and reads with reads falling below this level were omitted from error rate calculation for that position. C. The 3' error rates for the datasets as the Phred quality threshold is increased.

Table S1: Data sources. The wild type and deletion strain data included in this meta-analysis. All data were downloaded from the National Center for Biotechnology Informations Gene Expression Omnibus. The equivalent wild type RNA-seq data were also analyzed.

Species	Dataset	Platform	Accession	Ref
Saccharomyces cerevisiae	*Sc*WT<br>*Sc*RNA<br>*Sc*ΔTFIIS	Illumina Genome Analyser II	GSE25107	[1]
Escherichia coli	*Ec*WT<br>*Ec*RNA<br>*Ec*ΔGre	Illumina HiSeq 2000	GSE56720	[2]

Table S2: Accuracy of error rates. False positive error rates for the reverse transcriptase (RT), polymerase chain reaction (PCR) and sequencing (SEQ) stages of the NET-seq protocol. RT and PCR rates are calculated based on the manufacturers reported error rates while sequencing error rates are calculated based on a Phred quality threshold of 30. Accuracy of the error rates was then calculated as the percentage of all observed misincorporations that were not attributable to experimental false positives.

**Strain**	**Observed error rate**		**Experimental error rate**			**Accuracy (%)**	
	**3**	**-1 to -10**	**RT**	**PCR**	**SEQ**	**3**	**-1 to -10**
*Sc*WT	1.11x10^{-2}	1.63x10^{-3}	6.5x10^{-5}	4.4x10^{-7}	1.0x10^{-3}	90.99	38.65
*Sc*ΔTFIIS	7.00x10^{-2}	4.33x10^{-3}				98.57	76.91
*Sc*RNA	3.59x10^{-3}	1.49x10^{-3}				72.14	32.89
*Ec*WT	2.81x10^{-2}	1.68x10^{-3}	6.5x10^{-5}	4.4x10^{-7}	1.0x10^{-3}	96.44	40.48
*Ec*ΔGre	5.73x10^{-2}	2.28x10^{-3}				98.25	56.14
*Ec*RNA	1.21x10^{-2}	2.32x10^{-3}				91.74	56.90

Table S3: *Saccharomyces cerevisiae* alignment statistics. The total numbers of reads aligning to the genome for the *S. cerevisiae* datasets while allowing one, two and three mismatches (mm) in the seed region of the alignment. The number of reads aligning to RNA are also displayed.

**Dataset**	**Reads**	**tRNA**	**snoRNA**	**rRNA**	**# mm**	**Aligned**
*Sc*WT	63709986	594958	419395	30469062	1	18007915
		0.93%	0.66%	47.82%		28.27%
					2	18134305
						28.46%
					3	18134233
						28.46%
*Sc*ΔTFIIS	50177404	372206	667670	21272496	1	11183635
		0.74%	1.33%	42.39%		22.29%
					2	11401566
						22.72%
					3	11402017
						22.72%
*Sc*RNA	50898888	93999	18963	27160142	1	13399322
		0.18%	0.04%	53.36%		26.33%
					2	13471539
						26.47%
					3	13471624
						26.47%

Table S4: *Escherichia coli* alignment statistics. The total numbers of reads aligning to the genome for the *E. coli* datasets while allowing one, two and three mismatches (mm) in the seed region of the alignment.

Dataset	# mm	Total reads	Aligned	Percentage
*Ec*WT	1	66320440	35415011	53.40%
	2		35867134	54.08%
	3		35892771	54.12%
*Ec*ΔGre	1	42929547	22163387	51.63%
	2		22371035	52.11%
	3		22373984	52.12%
*Ec*RNA	1	38645886	5716198	14.79%
	2		5752800	14.89%
	3		5753490	14.89%

References

[1] Churchman, L.S. & Weissman, J.S. Nascent transcript sequencing visualizes transcription at nucleotide resolution. *Nature*, 2011, 469, 368-373. doi:10.1038/nature09652.

[2] Larson, M.H., Mooney, R.A., Peters, J.M., Windgassen, T., Nayak, D., Gross, C.A., Block, S.M., Greenleaf, W.J., Landick, R. & Weissman, J.S. A pause sequence enriched at translation start sites drives transcription dynamics *in vivo* *Science*, 2014, 344, 1042-1047. doi:10.1126/science.1251871.
